# Global biogeography of warning coloration in the butterfly *Danaus chrysippus*

**DOI:** 10.1098/rsbl.2021.0639

**Published:** 2022-06-01

**Authors:** Wanzhen Liu, David A. S. Smith, Gayatri Raina, Rowan Stanforth, Ivy Ng'Iru, Piera Ireri, Dino J. Martins, Ian J. Gordon, Simon H. Martin

**Affiliations:** ^1^ College of Life Sciences, South China Agricultural University, Guangzhou, Guangdong, People's Republic of China; ^2^ Natural History Museum, Eton College, Windsor, UK; ^3^ Department of Biological Sciences, Indian Institute of Science Education and Research, Mohali, India; ^4^ School of Biology, University of St Andrews, St Andrews, UK; ^5^ Mpala Research Centre, Nanyuki, Kenya; ^6^ International Centre for Insect Physiology and Ecology, Nairobi, Kenya; ^7^ Department of Ecology and Evolutionary Biology, Princeton University, Princeton, NJ, USA; ^8^ Centre of Excellence in Biodiversity and Natural Resource Management, University of Rwanda Huye Campus, Huye, Rwanda; ^9^ Institute of Evolutionary Biology, University of Edinburgh, Edinburgh, UK

**Keywords:** polymorphism, clines, dispersal, citizen science, aposematic

## Abstract

Warning coloration provides a textbook example of natural selection, but the frequent observation of polymorphism in aposematic species presents an evolutionary puzzle. We investigated biogeography and polymorphism of warning patterns in the widespread butterfly *Danaus chrysippus* using records from citizen science (*n* = 5467), museums (*n* = 8864) and fieldwork (*n* = 2586). We find that polymorphism in three traits controlled by known mendelian loci is extensive. Broad allele frequency clines, hundreds of kilometres wide, suggest a balance between long-range dispersal and predation of unfamiliar morphs. Mismatched clines for the white hindwing and forewing tip in East Africa are consistent with a previous finding that the black wingtip allele has spread recently in the region through hitchhiking with a heritable endosymbiont. Light/dark background coloration shows more extensive polymorphism. The darker genotype is more common in cooler regions, possibly reflecting a trade-off between thermoregulation and predator warning. Overall, our findings show how studying local adaptation at the global scale provides a more complete picture of the evolutionary forces involved.

## 1. Introduction

Warning coloration in chemically protected species provides some of the best-studied examples of natural selection. Learned predator avoidance should select for the most common warning pattern [1]. It is therefore intriguing that many aposematic species are polymorphic or geographically variable [2]. This may reflect neutral processes such as dispersal and shifting balance [3], or opposing forces such as sexual selection [4] and overdominance [5]. When warning patterns vary geographically, selection can restrict regions of polymorphism to narrow hybrid zones with sharp allele-frequency clines (e.g. 5–20 km wide in the butterfly *Heliconius erato* [6]). However, biogeographic studies such as those cited above are usually restricted to a limited number of sampling sites or transects, impeding our ability to understand the full range of evolutionary forces involved.

**Figure 1.**
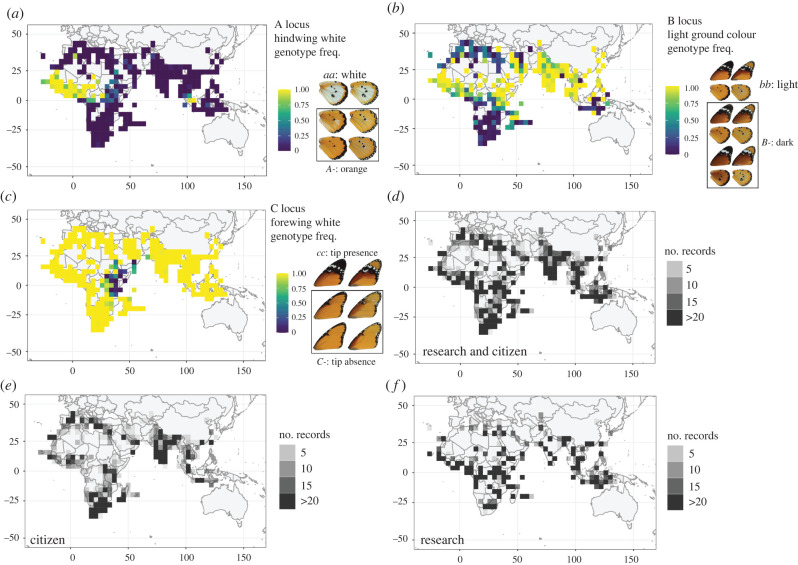
Colour pattern genotype frequency distributions (*a–c*) and number of records (*d–f*) based on 17 094 records. Each square represents a 4 × 4 degree grid cell and its colour represents the frequency of the homozygous recessive genotype (*a–c*) or number of records (*d–f*). Heterozygous and dominant homozygous genotypes were combined as their phenotypes are often indistinguishable (electronic supplementary material, figure S1).

Here we study the dispersive, chemically protected butterfly *Danaus chrysippus* (known as the African monarch, African queen or plain tiger). Several *D. chrysippus* morphs with distinct colour patterns are considered geographical subspecies or ‘semispecies’, with the latter term used to acknowledge the extensive range overlap seen in a broad polymorphic ‘hybrid zone’ in east Africa [7]. The colour variation is controlled by three genetic loci called A, B and C (figure 1 and electronic supplementary material, S1), with B and C being linked together in a supergene [8–11]. Genetic differentiation is very low across the range, with the exception of a few genomic ‘islands of divergence’, including the A, B and C loci [9]. This suggests that, despite the extensive polymorphism, warning pattern differences between regions are maintained by selection. A previous study found evidence for allele frequency clines in east Africa, but their vast geographical extent and limited sampling sites prevented detailed cline analysis [7].

We explored how selection acts on colour variation across the entire range of *D. chrysippus* by combining conventional research data (museum and field collections/observations) with citizen science data from online biodiversity recording platforms. We first assessed the feasibility of citizen science data for reliable scoring and mapping of trait variation. We then quantified polymorphism and asked whether allele frequencies vary clinally through space, comparing clines for different loci. Finally, we tested whether melanic background coloration (B locus) is associated with abiotic environmental variables.

## 2. Methods

### (a) Data sources

We considered two sources of *D. chrysippus* records (electronic supplementary material, table S1). ‘Research records’ included museum collections and field collections/observations [7,9]. ‘Citizen science records' are those from online biodiversity monitoring platforms to which users can upload photographs and other metadata. These were obtained from the Global Biodiversity Information Facility (GBIF), which collects observations from multiple online databases (electronic supplementary material, table S1, last downloaded on 1 November 2021, https://doi.org/10.15468/dl.8ugwwt).

### (b) Phenotyping and allele frequency estimation

We scored each butterfly for three traits, corresponding to three known loci: hindwing white/orange (A locus), light orange/dark brown background colour (B locus) and presence/absence of forewing black tip (C locus) (figure 1, electronic supplementary material, S1). Heterozygotes at all three loci are not reliably scorable due to variable penetrance [12]. We, therefore, considered two genotype classes: homozygous recessive (e.g. *aa*) and dominant (e.g. *A-*) (figure 1, electronic supplementary material, S1). Records were grouped in grid cells of 4 × 4 or 2 × 2 degrees (latitude and longitude) and allele frequencies were estimated assuming Hardy–Weinberg equilibrium. Maps were plotted using the R packages ggplot2, maps and mapproj [13–16].

### (c) Transects and cline analysis

We selected four transects that connected approximate regions of highest abundance of contrasting genotypes at two of the three loci. For cline analysis, we used the estimated allele frequencies for each grid cell within 450 km of the transect path, accounting for the curvature of the earth (cross-track distance). Position along the transect for each cell was the position nearest to the centre of the cell (along-track distance). Distances were calculated using the R package geosphere [17]. We used HZAR [18] to fit cline models, and compared the fits with different parameters for scaling (none, fixed, free) and tails (none, both) using corrected AIC scores. Cline parameters were estimated using 10^5^ generations of an MCMC search in three independent chains after 10^4^ burn-in generations.

### (d) Environmental associations

To test the hypothesis that the frequency of dark/light morphs (B locus) may be partly driven by the abiotic environment [19], we selected four environmental variables of potential relevance from the CliMond database [20] (averaged 1961–1990): annual mean temperature (bio01), solar radiation (bio20), annual precipitation (bio12) and soil moisture index (bio28) (electronic supplementary material, figure S10–S11). Variable layers were processed and checked for errors using Arcmap v. 10.3 (ESRI, 2015). We extracted the corresponding variables for each butterfly record location using the R packages rgdal, rgeos and maps [13,21,22]. For records where coordinates were out of range of the variable layers (*N* = 283) we manually applied values of nearby areas.

To test for associations, we first assessed Pearson correlations and pairwise linear regressions between each environmental variable (averaged across all record locations within each grid cell) and genotype frequency, weighted by the number of records per cell. We then generated generalized least-squares (GLS) models using the R package nlme [23]: one null model and 15 models including all combinations of explanatory variables. The GLS accounted for spatial autocorrelation assuming that errors are exponentially related to the Euclidean distance between grid cells based on coordinates. Variance weights were defined as the inverse of the number of records per grid cell. We assessed the effect of correlation among the explanatory variables using the PerformanceAnalytics R package and by quantifying the variance inflation factor (VIF) [24,25].

## 3. Results

### (a) Citizen science data fills gaps in research collections

We describe distributions of *D. chrysippus* colour pattern traits based on 17 094 records (figure 1). Although citizen science records represent a third of the dataset, they cover 83% of grid cells (228/275) compared with 53% (147) covered by the research data (figure 1*d–f*). Genotype frequencies in the research data were strongly predictive of the citizen science data for forewing tip (C locus, *R*^2^ = 0.887, *p* < 2.2 × 10^−16^), and hindwing white (A locus, *R*^2^ = 0.7109, *p* < 2.2 × 10^−16^), but less so for background colour (B locus, *R*^2^ = 0.367, *p* = 6.02 × 10^−10^) (electronic supplementary material, figure S2). This weaker correlation is partly due to the fact that background colour could not be scored from images of wing undersides (approx. 50% of citizen science records). However, it may also indicate biological change over time, as citizen science records tend to be more recent.

Distributions of the three traits largely agree with previous hand-drawn maps [26], with one major exception: dark background colour (*B-*) is common in southern Europe and North Africa (figure 1*b*). It is unclear whether this represents a change in genotype frequency because historic records for this region are limited. One notable change over time is the hotspot of the white hindwing (*aa*) in southeast Asia (figure 1*a*), which arose recently [27].

### (b) Extensive polymorphism

All three traits are polymorphic over a considerable proportion of the range. The absence of a forewing black tip (*C-* genotype) approaches fixation (frequency greater than 0.95) in just 2.5% of grid cells, but is polymorphic (≥0.05 and ≤0.95) in 9% of cells. This low level of fixation partly reflects poor representation in the heart of the range of this phenotype in the horn of Africa (figure 1*c*, electronic supplementary material, S2). White hindwing patch (*aa*) approaches fixation in 9% of cells but is polymorphic in 16%. Dark background colour (*B-*) has the broadest and patchiest distribution, approaching fixation in 17% but polymorphic in 45%.

### (c) Broad allele frequency clines

**Figure 2.**
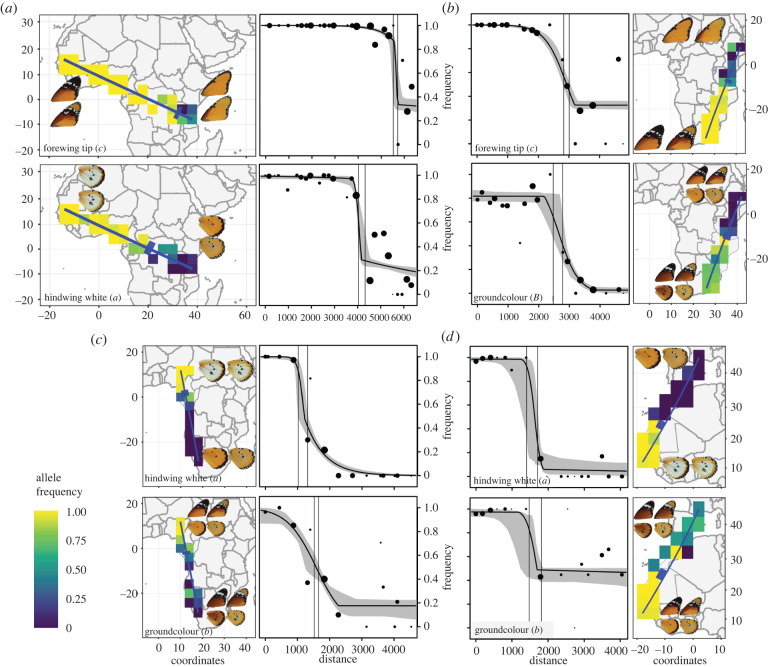
Transects and allele frequency clines. Each point along the transect represents a grid cell within 450 km from the transect, with the size of the point drawn relative to the number of records. The solid line is the best-fit model, and the surrounding grey region represents the 95% credible fuzzy cline region. Two solid vertical lines represent the cline centre confidence interval (details in electronic supplementary material, table S2). In all cases, the frequency represents the recessive allele (*a*, *b* or *c*), except for the ground colour locus in panel *b*, where the frequency of the dominant allele (*B*) is shown for convenience. Map inserts show the grid cells included and their corresponding frequencies. The confidence interval for cline centres are indicated by blue boxes.

Allele frequency clines along four transects in Africa range from approximately 290 to approximately 1800 km in width, and tend to be narrower for hindwing white (A locus) and broadest for background colour (B locus) (figure 2*a–d* and table 1). In three of the four transects, cline centres are similar for the two traits considered (figure 2*b–d*). Coincident clines are expected for the B and C loci (southeast African transect) due to linkage [9]. By contrast, the nearly identical cline centres for the unlinked A and B loci in northwest Africa (figure 2*d*), suggest concordant selection pressures on both traits and/or a biogeographic break. Clines along the central African transect are strongly mismatched, with the forewing tip (C) cline centre approximately 1500 km east of the hindwing white (A) cline centre (figure 2*a* and table 1). The cline fitting results remain consistent when smaller grid cells and broader transects are used (electronic supplementary material, figure S3–S6).

**Table 1.** Cline parameters estimated under model 6 (scaling = fixed, tails = both).

transect	no. locations	no. records	cline width (km)			cline centre offset (km)^a^
			hindwing white (A locus)	ground colour (B locus)	forewing tip (C locus)	
Central Africa	25	3809	427 (216–968)		290 (111–623)	1551 (1506–1398)
Southeast Africa	19	2436		1252 (631–628)	1472 (1003–1636)	260 (373–252)
Southwest Africa	15	1205	411 (27–563)	1781 (1547–2151)		352 (509–346)
Northwest Africa	17	612	349 (167–859)	764 (358–1672)		40 (23–110)

^a^Cline centre offset indicates the difference in cline centre (average, lowest and highest) between the two loci considered in each transect. See electronic supplementary material, table S2 for details.

### (d) Darker background colour is more frequent in cooler regions

We used regression analyses to test for an association between wing background colour (B locus) and abiotic environmental variables. These reveal a strong relationship with annual mean temperature, with a higher frequency of darker morphs in cooler regions (electronic supplementary material, figure S8–S9 and table S3–S4). We fitted GLS models including four environmental variables and all combinations (electronic supplementary material, table S3–S4, figures S7–S9). The best fit model includes annual mean temperature, annual precipitation and soil moisture index. However, only annual mean temperature meets all of the following criteria: (i) is included in all of the top five best-fitting models, (ii) has an effect size significantly different from zero and (iii) is also significant in a linear model (*p* = 1.46 × 10^−7^) and unweighted Pearson correlation (*p* = 6.1 × 10^−10^) (electronic supplementary material, table S3–S4, figure S8–S9). Annual precipitation and soil moisture index showed strong collinearity with each other (VIF greater than 11 and Pearson coefficient greater than 0.9, electronic supplementary material, table S3c), which might have inflated their fit in the best model. GLS models fitted using smaller 2 × 2 degree grid cells produce very similar results (electronic supplementary material, table S3–S4). Overall, these results imply that temperature is a likely driver of selection on wing melanism. Maps of the trait frequency and annual mean temperature are visually similar (electronic supplementary material, figure S10, S11).

## 4. Discussion

By combining research collections and citizen science data, we were able to study variation in warning colour in *D. chrysippus* at the global scale. The clinal variation is consistent with a balance between selection and dispersal, but the scale of these clines is one or two orders of magnitude greater than those in other warningly coloured butterflies [6], implying long-distance dispersal, weak selection or both. The extraordinary dispersal ability of *D. chrysippus* is evidenced by its occurrence on nearly every island in the Indian and southern Atlantic oceans [28] and an absence of genetic differentiation between distant populations [9]. Similar cline centres for unlinked loci support a model in which predator learning maintains morph distributions by selecting against rare forms [6,29,30]. However, the stark offset between clines in central to eastern Africa suggests that other evolutionary forces are at play. A previous genomic study revealed that the recessive black wingtip allele (*c*), has spread recently in East Africa by hitchhiking with a male-killing endosymbiont [9]. This likely accounts for the eastward shift of this cline relative to that of the white hindwing (*a*) allele. The true offset between these clines may be underestimated due to our assumption of Hardy–Weinberg equilibrium. Immigration can cause an excess of homozygotes, which would lead us to overestimate the range of the recessive white hindwing (*a*) allele. By contrast, there is a known excess of *Cc* heterozygotes due to male-killing [7], which would lead to an underestimate of the full extent of the eastward shift of the recessive black wingtip (*c*) allele. A targeted cline analysis using genetic data could verify this hypothesis.

In addition to predator warning, wing coloration—specifically light/dark wing background controlled by the B locus, which is associated with the melanin pathway gene yellow [9]—appears to be partly driven by abiotic environmental selection. Darker coloration is associated with cooler regions, following a trend also seen across butterfly species [19]. This likely explains the enhanced polymorphism at the B locus. Whether selection for thermoregulation leads to a trade-off with warning coloration [31] remains to be tested.

Citizen science and research records have different merits [32]. In our dataset, citizen science records tended to have more reliable locality data than museum specimens, but the associated images were often insufficient to score all three traits. Both data sources suffer from geographical bias in sampling effort (less so for citizen science), but this is unlikely to significantly impact our results, as we used the relative frequencies of genotypes rather than absolute abundance. Future studies could improve on our approach by using two-dimensional models of allele frequency change through space, as well as automated detection of phenotypes and other ‘secondary data’ [33] from images in the ever-growing citizen science databases.

## Data Availability

All raw data and scripts to perform all analyses, along with a description of the data and scripts, are available from the Dryad Digital Repository: https://doi.org/10.5061/dryad.j9kd51cfm [34]. The data are provided in electronic supplementary material [35].
